# Hereditary genetic testing and mainstreaming: a guide for surgeons

**DOI:** 10.1308/rcsann.2024.0029

**Published:** 2024-04-01

**Authors:** A Monaghan, E Copson, R Cutress

**Affiliations:** ^1^ University Hospital Southampton NHS Foundation Trust, UK; ^2^ University of Southampton, UK

Since the discovery of the *BRCA1* and *BRCA2* genes, and their association with breast cancer in 1994, their clinical impact and indeed public notability have increased exponentially. Traditionally, the provision of testing for so-called cancer-predisposing germline pathogenic variants (GPVs) or hereditary genetic mutations, for example in *BRCA* genes, has always been the purview of clinical genetics departments alone. However, due to rising referrals for such tests and the increased affordability of the relevant technologies, demand is now very much outstripping the current system’s capacity.^1,2^

In addition, the importance of germline testing with regard to treatment planning is also becoming progressively apparent. Various interventions, including both risk-reducing (prophylactic) surgery^3^ and, more recently, the use of poly(ADP-ribose) polymerase (PARP) inhibitors (e.g. olaparib), are influenced heavily by patients’ *BRCA* status. Consequently, surgeons now have more of a direct interest in ensuring timely testing for their patients and in accessing their results promptly. These changes are ushering in a new paradigm for the structure of *BRCA* testing, which accounts for the evolving clinical landscape.

This ‘mainstreaming’ approach is proposed as a potential means of addressing the current system’s shortcomings, for example in cases where there is a confirmed cancer diagnosis. It moves responsibility for some aspects of the genetic testing process to other professionals involved in the patient’s clinical care such as their surgeon or oncologist.^1,2^ For this reason, it is becoming increasingly important that these clinicians are well versed in the underlying genomic science, the structure of the testing system, and the ramifications of results for prognostication and planning of management, be it risk-reducing or therapeutic. Consistent with this, surgeons have now been added to the list of requesting specialties for a variety of germline genetic tests across a range of indications in the national guidance (Table 1).^4^

**Table 1:** Summary of genomic testing for which surgeons or surgical specialties are listed as requesting clinicians in the NHS national genomic test directory^4^

**Surgical specialty**	**Gene target(s)**	**Criteria reference**	**Condition**	General clinical circumstances on which testing criteria are based*
Colorectal and gynaecological	*MLH1*, *MSH2*, *MSH6*, *PMS2*	R210	Lynch syndrome	DNA mismatch repair colorectal or endometrial tumour and/or relevant personal/family history
Colorectal	Inherited polyposis small gene panel (currently *APC*, *BMPR1A*, *EPCAM*, *MLH1*, *MSH2*, *MSH6*, *MUTYH*, *NTHL1*, *PMS2*, *POLD1*, *POLE*, *PTEN*, *RNF43*, *SMAD4*, *STK11*)	R211	Inherited polyposis	Appropriate personal/family history of polyps/early-onset colorectal cancer
Gynaecological and obstetric	*CFTR*	R184	CF	Obstructive azoospermia with absence of vas deferens, or fetal features suggestive of CF
Gynaecological	*CFTR*	R185	CF carrier testing	Egg or sperm donor, family history of CF or both parents of fetus with suspected CF
Gynaecological	*BRCA1*, *BRCA2*, *BRIP1*, *MLH1*, *MSH2*, *MSH6*, *PALB2*, *RAD51C*, *RAD51D*	R207	Ovarian cancer	Appropriate personal/family history of ovarian cancer (without breast cancer)
Gynaecological	Karyotype, *FMR1*	R402	Premature ovarian insufficiency	Personal history of premature ovarian insufficiency with biochemical confirmation
Neurosurgery and urology	*VHL*	R225	VHL disease	Appropriate personal/family history of VHL-related tumours (e.g. cerebellar haemangioblastoma &lt;60 years)
Neurosurgery	*SMAD6*	R416	Craniosynostosis	Confirmed craniosynostosis
Orthopaedic	*EXT1*, *EXT2*	R390	Osteochondroma	History of multiple exostoses
Paediatric	*RET*	R177	Hirschsprung’s disease	Intestinal failure and/or short bowel syndrome in childhood
Plastic surgery	Segmental overgrowth small gene panel (currently *AKT1*, *AKT3*, *CCND2*, *CDKN1C*, *PADI6*, *PIK3CA*, *PIK3R2*, *PTEN*, *RASA1*, *SUZ12*)	R110	Segmental overgrowth disorders	Appropriate features that may include megalencephaly, vascular malformations and cutaneous features
Surgery (subspecialty not specified)	*ATM*, *BRCA1*, *BRCA2*, *CHEK2*, *PALB2*, *RAD51C*, *RAD51D*	R208	Inherited breast/ovarian cancer	Appropriate personal/family history of breast/ovarian cancer
Surgery (subspecialty not specified)	*STK11*	R212	Peutz–Jeghers syndrome	Appropriate personal/family history of hamartomatous polyps and/or Peutz–Jeghers syndrome features
Surgery (subspecialty not specified)	*APC*	R414	*APC*-associated polyposis	Children/young adults with *APC*-associated features
Surgery (subspecialty not specified)	*BRCA1*; *BRCA2*	R444	Inherited breast cancer	Patients not meeting R208 criteria AND with current cancer diagnosis for treatment decisions AND meeting NICE criteria for adjuvant PARP inhibitor treatment
Surgical dentistry	Ectodermal dysplasia medium gene panel or WES (see PanelApp for current list)^27^	R163	Ectodermal dysplasia	Appropriate abnormalities of hair, teeth or skin
Surgical dentistry	Amelogenesis imperfecta medium gene panel or WES (see PanelApp for current list)^27^	R340	Amelogenesis imperfecta	Enamel abnormalities
Upper gastrointestinal	*CDH1*, *CTNNA1*	R215	Gastric cancer	Appropriate personal/family history of gastric/lobular breast cancer
Urology	*BAP1*, *FH*, *FLCN*, *MET, SDHB*, *VHL*	R224	Renal cell cancer	Appropriate personal/family history of renal cancer/associated inherited cancer syndrome
Urology	*FH*	R365	Fumarate hydratase-related tumour syndromes	Personal history of hereditary leiomyomatosis and renal cell cancer meeting appropriate criteria
Urology	Y chromosome microdeletions	R411	Infertility	Male infertility
Urology	*ATM*, *BRCA1*, *BRCA2*, *CHEK2*, *MLH1*, *MSH2*, *MSH6*, *PALB2*	R430	Prostate cancer	Appropriate personal/family history of prostate cancer
Urology	*BRCA1*, *BRCA2*	R444	Prostate cancer	Metastatic, castration-resistant prostate cancer where somatic tumour testing (M218.1) has failed
CF = cystic fibrosis; NICE = National Institute for Health and Care Excellence; PARP = poly(ADP-ribose) polymerase; VHL = Von Hippel–Lindau; WES = whole exome sequencing. *The specific details and criteria are found in the test directory itself.^4^				

## Why mainstreaming?

In practice, a mainstreamed framework would require surgeons to take partial responsibility when assessing eligibility for and arranging germline genetic testing of cancer-susceptibility genes in affected patients. It is argued that streamlining in this way would improve the efficiency of the system overall by curbing the instances of seemingly inexorable delays typically associated with interspecialty referrals. This, it is hoped, will lead to shorter referral times for tests, quicker turnarounds for results and ultimately, a more cost effective structure with improved outcomes for patients.^1^

The process would also inevitably involve discussions between surgeons and patients on the utility of testing, and the significance of a positive result. In this model, only those who went on to test positive for pathological variants or those found to carry a variant of uncertain clinical significance (VUS) would be referred to clinical genetics specialists for further counselling and management planning.^1^ It is anticipated that this will help relieve the pressure on these departments by limiting their involvement to only patients with confirmed cancer-predisposing GPVs, on whom their expertise is best focused. The *quid pro quo* for surgeons would be facilitating faster and more focused testing, the results of which are likely to have a direct impact on the treatments they recommend.

## Assessing eligibility for germline genetic testing (exemplified by *BRCA* testing in breast cancer)

Current National Institute for Health and Care Excellence guidelines suggest that breast cancer patients are candidates for germline *BRCA* testing if they are deemed to have a risk of 10% or more of being a carrier of a *BRCA1* or *BRCA2* mutation.^5^ This risk can be determined using a verified scoring system such as the Manchester criteria^5,6^ or BOADICEA (Breast and Ovarian Analysis of Disease Incidence and Carrier Estimation Algorithm),^5,7^ both of which approximate risk based primarily on family history. The online CanRisk tool from the University of Cambridge is a user-friendly calculator based on the BOADICEA model, which clinicians may find helpful and can be accessed after completing the new user registration form on the website.^8^

Additional, more detailed eligibility criteria are listed in the national genomic test directory (section R208).^4^ These are not intended specifically for mainstreaming but it is likely that the ultimate criteria used will be based (at least partly) on this. The feasibility of mainstreaming in germline *BRCA* testing was first demonstrated by the Mainstreaming Cancer Genetics programme, which initially proposed a simplified set of criteria.^9^ Based at the Royal Marsden Hospital in London, this group pioneered the use of mainstreaming in breast and ovarian cancer patients using these criteria for germline *BRCA* testing specifically intended to be more accessible and easily practicable for non-genetics specialists. The validity of their eligibility criteria was substantiated by a three-year prospective study, which demonstrated a 10% detection rate of *BRCA* variants in addition to good cost and time efficiency.^10^

Criteria for germline *BRCA* testing have evolved over recent years and since 2020, the national genomic test directory has dictated testing standards for breast cancer-predisposing GPVs throughout the NHS in England.^4^ For ease of practice, some breast cancer units have now agreed that patients must meet one of the first four or five R208 criteria for mainstreaming purposes (Table 2), with patients requiring more complex genetic risk assessments still being referred to clinical genetics departments. Eligibility criteria in the devolved nations varies by geographical location.

**Table 2:** Eligibility criteria for germline testing of breast cancer-susceptibility genes^4^

R208 criteria for germline testing in those with a personal history of breast cancer (invasive or high-grade DCIS) or high-grade ovarian cancer	Germline (heritable) testing of the following genes:
Breast cancer &lt;40 years OR Bilateral breast cancer &lt;50 years OR Triple negative breast cancer &lt;60 years OR Biological male affected with breast cancer (any age) OR Breast cancer &lt;45 years AND first-degree relative with breast cancer &lt;45 years OR Combined pathology-adjusted Manchester score ≥15 or single gene pathology-adjusted score ≥10 or BOADICEA/CanRisk score ≥10% OR Ashkenazi Jewish ancestry and breast cancer at any age	*BRCA1* *BRCA2* *PALB2* ATM* CHEK2* RAD51C* RAD51D* *truncating variants only
BOADICEA = Breast and Ovarian Analysis of Disease Incidence and Carrier Estimation Algorithm; DCIS = ductal carcinoma in situ	

A more recent study, BRCA-DIRECT, has trialled the use of a rapid, digital pathway supported by a specialist genetics hotline, which may represent mainstreaming’s future direction. Pilot data indicate high uptake of genetic testing and good satisfaction.^11^ As alluded to already, the national genomic test directory R208 guidance lists surgeons as one of the requesting specialties for testing (along with oncologists and geneticists),^4^ paving the way for mainstreamed practice.

## Preparing for mainstreaming

How can surgeons prepare for this new era? While mainstreaming has been introduced in some areas with minimal reported impacts on capacity, some feel that rather than surgeons themselves shouldering this increased work burden, mainstreaming is best enacted through the establishment of a separate specialist nurse role to run specific mainstreamed clinics. This has been trialled in some centres with success,^12^ and it is possible that this offers a compromise that best serves both the interests of patients and the surgeons themselves. It may be simply unrealistic to expect surgeons (or oncologists or other clinicians outside genetic services) to make time in their clinics to have discussions handled previously by an entirely different, dedicated department. If this is to be the way forwards, however, business cases may be required to justify this new role and secure funding.

In any case, bringing genetic testing further upstream in the clinical timeline will inevitably require surgeons to have more involvement in (and knowledge of) the testing process than before. Its implications for risk-reducing surgery (including in unaffected relatives who are found to carry GPVs)^3^ also mean that discussions around genetic status are increasingly likely to form at least part of surgeons’ consultations in future.

As mentioned, a key obstacle will be in counselling patients proactively on the need for genetic investigations and the significance of potential outcomes as this pertains to future risk and prophylactic interventions. This is outside of surgeons’ usual scope of practice and indeed, evidence shows that it is one of their chief objections to embracing mainstreaming.^13,14^ Understanding the fundamental genetic principles underpinning GPVs is one thing but many surgeons may feel unequipped to discuss the nuances of personal and hereditary risk associated with various test results in a way that is accessible and patient-centred. After all, this has traditionally been the role of specially trained genetics counsellors or clinical geneticists themselves.

One key attempt to address this in breast cancer is the Mainstreaming Cancer Genetics programme’s own *BRCA* toolkit.^9^ This learning resource consists of online videos, presentations and answers to frequently asked questions, which ensures complete familiarity with the mainstreamed testing process and even provides a downloadable certificate on completion. The *BRCA* toolkit was used as the primary training method at Imperial College Healthcare NHS Trust when mainstreaming was introduced there, and led to a successful implementation with significantly improved testing uptake and turnaround times.^15^ Other trusts have opted to produce their own e-modules, such as at Cambridge University Hospitals.^16^ Furthermore, Health Education England’s GeNotes is a valuable aid for reacquainting clinicians with the salient genetics knowledge and relevant referral criteria to allow efficacious mainstreamed practice.^17^

For those seeking more intensive training, another more hands-on training course has emerged in the form of the TRUSTING (Talking about Risk, UncertaintieS of Testing IN Genetics) workshop.^18^ This two-day programme seeks to demystify pertinent conversations around cancer-susceptibility gene testing (with a particular focus on *BRCA* testing), and to give clinicians the tools to discuss these issues with their patients confidently and effectively. This training is seen by its organisers as an essential prerequisite to facilitating mainstreaming, and has been shown to be successful in improving knowledge, communication skills and self-confidence in a cohort of delegates, 21 of whom were surgeons.^19^ Similar, smaller-scale training courses also exist in many centres where mainstreaming is already being introduced such as in Nottingham.^20^

## Evaluating mainstreaming and future perspectives

Surgeons’ understandable caution regarding mainstreamed genetic testing is well documented^21^ and this model will of course require careful implementation. In addition to the communication challenges highlighted previously, surgeons may also perceive assuming this additional responsibility as unsustainable^14,21^ and as simply representing a redistribution of current workload rather than an overall efficiency-saving change. As discussed, some may feel that this is best addressed by the formation of an entirely new specialist nurse role. A further barrier is the complexity associated with variants of uncertain clinical significance^13^ although this can be remedied easily by ensuring that detection of patients with such variants triggers a referral to genetics services in the same way known pathogenic variants do.

Despite mainstreaming’s potential pitfalls, it appears inescapable that the nature of germline genetic testing in the UK is moving towards direct access to treating clinicians. In many regions, *BRCA* testing in ovarian cancer patients is already mainstreamed owing to the importance of prompt *BRCA* status characterisation for the commencement of PARP inhibitors such as olaparib in affected patients.^12,22,23^ Notably, the seminal OlympiA study has more recently demonstrated the benefit of adjuvant olaparib treatment in *BRCA*-positive early breast cancer patients as well,^24^ further underlining the need for expedited, widely available gene testing. Discussions around the mainstreaming of gene testing in colorectal cancer patients (e.g. Lynch syndrome/hereditary non-polyposis colorectal cancer) are also gaining momentum.^25,26^

While teething difficulties are foreseeable and perhaps inevitable, time will tell whether mainstreaming’s purported benefits are borne out by the data. Many, however, see this system as the future of *BRCA* testing for breast cancer patients and widening of clinician access to hereditary genetic testing more generally as the future direction nationally.

## References

[1] Rahman N. Mainstreaming genetic testing of cancer predisposition genes. *Clin Med* 2014; **14**: 436–439. 10.7861/clinmedicine.14-4-436 PMC4312836 25099850

[2] Slade I, Riddell D, Turnbull C *et al.* Development of cancer genetic services in the UK: a national consultation. *Genome Med* 2015; **7**: 18. 10.1186/s13073-015-0128-4 PMC4341881 25722743

[3] McCarthy RL, Copson E, Tapper W *et al.* Risk-reducing surgery for individuals with cancer-predisposing germline pathogenic variants and no personal cancer history: a review of current UK guidelines. *Br J Cancer* 2023; **129**: 383–392. 37258796 10.1038/s41416-023-02296-w PMC10403612

[4] NHS England. National genomic test directory. https://www.england.nhs.uk/wp-content/uploads/2018/08/Rare-and-inherited-disease-eligibility-criteria-version-6-January-2024.pdf (cited March 2024).

[5] National Institute for Health and Care Excellence. *Familial Breast Cancer: Classification, Care and Managing Breast Cancer and Related Risks in People With a Family History of Breast Cancer (CG164)*. London: NICE; 2013. 31940157

[6] Evans DG, Eccles DM, Rahman N *et al*. A new scoring system for the chances of identifying a BRCA1/2 mutation outperforms existing models including BRCAPRO. *J Med Genet* 2004; **41**: 474–480. 15173236 10.1136/jmg.2003.017996 PMC1735807

[7] Antoniou AC, Pharoah PP, Smith P, Easton DF*.* The BOADICEA model of genetic susceptibility to breast and ovarian cancer. *Br J Cancer* 2004; **91**: 1 580–1 590. 15381934 10.1038/sj.bjc.6602175 PMC2409934

[8] CanRisk. New user registration form. https://www.canrisk.org/accounts/register/ (cited March 2024).

[9] Mainstreaming Cancer Genetics. What was the Mainstreaming Cancer Genetics programme? https://www.mcgprogramme.com/ (cited March 2024).

[10] Kemp Z, Turnbull A, Yost S *et al*. Evaluation of cancer-based criteria for use in mainstream BRCA1 and BRCA2 genetic testing in patients with breast cancer. *JAMA Netw Open* 2019; **2**: e194428. 10.1001/jamanetworkopen.2019.4428 PMC6632150 31125106

[11] Torr B, Jones C, Choi S *et al*. A digital pathway for genetic testing in UK NHS patients with cancer: BRCA-DIRECT randomised study internal pilot. *J Med Genet* 2022; **59**: 1 179–1 188. 10.1136/jmg-2022-108655 PMC9691828 35868849

[12] Percival N, George A, Gyertson J *et al*. The integration of BRCA testing into oncology clinics. *Br J Nurs* 2016; **25**: 690–694. 27345073 10.12968/bjon.2016.25.12.690 PMC5993185

[13] Lee YQ, Yoon SY, Hassan T *et al*. Attitudes and training needs of oncologists and surgeons in mainstreaming breast cancer genetic counseling in a low-to-middle income Asian country. *J Genet Couns* 2022; **31**: 1 080–1 089. 10.1002/jgc4.1579 35481858

[14] Hallowell N, Wright S, Stirling D *et al.* Moving into the mainstream: healthcare professionals’ views of implementing treatment focussed genetic testing in breast cancer care. *Fam Cancer* 2019; **18**: 293–301. 30689103 10.1007/s10689-019-00122-y PMC6560008

[15] Rumford M, Lythgoe M, McNeish I *et al.* Oncologist-led BRCA ‘mainstreaming’ in the ovarian cancer clinic: a study of 255 patients and its impact on their management. *Sci Rep* 2020; **10**: 3390. 10.1038/s41598-020-60149-5 PMC7042365 32098980

[16] NHS East Genomics. E-learning modules launched to help clinicians across the region order genomic tests for patients. https://www.eastgenomics.nhs.uk/about-us/news-and-events/new-e-learning-modules-launched-to-help-clinicians-across-the-region-order-genomic-tests-for-patients (cited March 2024).

[17] Health Education England. Welcome to GeNotes. https://genomicseducation.hee.nhs.uk/genotes/ (cited March 2024).

[18] SHORE-C. TRUSTING workshops 2024. https://shore-c.sussex.ac.uk/study.php?projid=128 (cited March 2024).

[19] Fallowfield L, Solis-Trapala I, Starkings R *et al.* Talking about Risk, UncertaintieS of Testing IN Genetics (TRUSTING): development and evaluation of an educational programme for healthcare professionals about BRCA1 & BRCA2 testing. *Br J Cancer* 2022; **127**: 1 116–1 122. 35715636 10.1038/s41416-022-01871-x PMC9470577

[20] Nottingham University Hospitals NHS Trust. Hereditary breast cancer. https://www.nuh.nhs.uk/hereditary-breast-cancer (cited March 2024).

[21] Wright S, Porteous M, Stirling D *et al*. Negotiating jurisdictional boundaries in response to new genetic possibilities in breast cancer care: the creation of an ‘oncogenetic taskscape’. *Soc Sci Med* 2019; **225**: 26–33. 30784848 10.1016/j.socscimed.2019.02.020

[22] Audeh MW, Carmichael J, Penson RT *et al*. Oral poly(ADP-ribose) polymerase inhibitor olaparib in patients with BRCA1 or BRCA2 mutations and recurrent ovarian cancer: a proof-of-concept trial. *Lancet* 2010; **376**: 245–251. 20609468 10.1016/S0140-6736(10)60893-8

[23] Moore K, Colombo N, Scambia G *et al*. Maintenance olaparib in patients with newly diagnosed advanced ovarian cancer. *N Engl J Med* 2018; **379**: 2 495–2 505. 10.1056/NEJMoa1810858 30345884

[24] Tutt AN, Garber JE, Kaufman B *et al*. Adjuvant olaparib for patients with BRCA1- or BRCA2-mutated breast cancer. *N Engl J Med* 2021; **384**: 2 394–2 405. 10.1056/NEJMoa2105215 PMC9126186 34081848

[25] Scheinberg T, Young A, Woo H *et al*. Mainstream consent programs for genetic counseling in cancer patients: a systematic review. *Asia Pac J Clin Oncol* 2021; **17**: 163–177. 32309911 10.1111/ajco.13334

[26] Georgiou D, Monje-Garcia L, Miles T *et al*. A focused clinical review of Lynch syndrome. *Cancer Manag Res* 2023; **15**: 67–85. 36699114 10.2147/CMAR.S283668 PMC9868283

[27] Genomics England. PanelApp. Genomics England PanelApp. https://panelapp.genomicsengland.co.uk/ (cited March 2024).

